# Job demand intensification and job satisfaction: engagement as a mediator

**DOI:** 10.3389/fpsyg.2026.1849644

**Published:** 2026-06-26

**Authors:** Kiralina Brito-Arancibia, Joan Boada-Grau, Beatriz Sora, Jean Paul Navarrete-Campos

**Affiliations:** 1 Department of Ergonomics, Faculty of Biological Sciences, University of Concepción, Concepción, Chile; 2 Departament of Psychology, Faculty of Psychology, Universitat Rovira i Virgili, Tarragona, Spain; 3 Department of Statistics, Faculty of Physical and Mathematical Sciences, University of Concepción, Concepción, Chile

**Keywords:** engagement, job demand intensification, job insecurity, job satisfaction, work–family conflict, emerging psychosocial risks

## Abstract

**Background:**

New psychosocial risks that challenge employees’ well-being and performance. This study investigates the mediating role of work engagement in the relationship between job demand intensification and job satisfaction, while accounting for job insecurity and work–family conflict as antecedents among Chilean workers.

**Methods:**

Data were collected from 420 employees in Chile in various economic sectors (62.1% women, 37.9% men; *M* age = 42.4, *SD* = 10.73) using validated instruments. Structural equation modeling was used to test the hypothesized model.

**Results:**

Job insecurity and work-to-family conflict significantly predicted job demand intensification. Engagement mediated the relationship between job demand intensification and job satisfaction, generating a positive indirect effect; in addition, job demand intensification showed a direct positive association with job satisfaction. Engagement emerged as a key motivational mechanism that channels intensified demands into greater satisfaction.

**Conclusion:**

Our findings advance the understanding of engagement as a mediating mechanism between job demand intensification and job satisfaction among Chilean workers. They highlight the organizational relevance of reducing job insecurity and work–family conflict, as well as fostering job conditions that sustain engagement under intensified work demands. These insights support the development of preventive and proactive initiatives to promote healthier work environments.

## 1 Introduction

Work is a fundamental human activity that mobilizes physical, cognitive, and emotional resources to produce goods and services, but also provides meaning, identity, and social integration (Neffa, 1998, 2023). Contemporary societies are undergoing profound transformations associated with technological innovation, globalization, and organizational restructuring, which accelerate the pace of life and reshape working conditions (Neffa, 2023; Rosa, 2003, 2015). These processes of social acceleration are generating new psychosocial risks that challenge employees’ well-being and performance (Correa and Quintero, 2020; Di Tecco et al., 2023). Among these, the intensification of job demands has emerged as a concern in occupational health research.

Job demand intensification refers to the requirement to perform an increasing number of tasks at a faster pace, often involving multitasking and reduced downtime. It has both quantitative (e.g., workload, time pressure) and qualitative (e.g., decision-making, learning demands) dimensions, making it a multifaceted phenomenon (Bunner et al., 2018; Green and McIntosh, 2001; Kubicek et al., 2014). Within the Job Demands–Resources (JD-R) model, and depending on available resources, demand intensification can function either as a hindrance, leading to strain, or as a challenge, stimulating growth and engagement (Bakker and Demerouti, 2007; Güngör and Sönmez, 2025).

Job insecurity constitutes another critical psychosocial risk that affects both individuals and organizations. It is defined as the perceived threat of job loss or the deterioration of valued job features and includes both cognitive evaluations of employment continuity and affective reactions such as anxiety or worry (Jiang and Lavaysse, 2018; Shoss, 2017). From the perspective of the Conservation of Resources (COR) theory (Hobfoll, 1989), job insecurity reflects a depletion of resources or an anticipation of future loss, thereby generating stress and undermining well-being. Evidence consistently shows that job insecurity reduces job satisfaction, organizational commitment, and psychological health (De Witte et al., 2016).

Work–family conflict is another well-established determinant of occupational health. It arises when demands from work and family are mutually incompatible, creating strain in both directions, with work interfering with family and family interfering with work (Frone, 2003; Greenhaus and Beutell, 1985). This bidirectional conflict has consistently been linked to higher stress, lower satisfaction, and lower engagement (Allen et al., 2000). Empirical evidence indicates that intensified job demands exacerbate this conflict by increasing work hours, fostering multitasking, and expanding the digital overlap between work and personal domains (Kubicek et al., 2015). The COVID-19 pandemic further illustrated these tensions: remote work often amplified work–family conflict, particularly among women, who reported difficulties balancing professional and domestic responsibilities along with increased loneliness and reduced productivity (Lyzwinski, 2024).

In contrast to these stressors, work engagement represents a positive motivational state characterized by vigor, dedication, and absorption in which people feel capable of coping effectively with job demands (Bakker et al., 2008; Hakanen and Schaufeli, 2012; Schaufeli and Bakker, 2010; Schaufeli et al., 2002). Engagement is regarded as a positive state of well-being, not merely the absence of tension (Schaufeli and Bakker, 2010). Engaged employees invest their physical, cognitive, and emotional energies in their roles, perceiving work as meaningful and stimulating (Kahn, 1990). Vigor is characterized by high levels of energy and resilience, reflected in the capacity to sustain effort. Dedication is characterized by enthusiasm, pride, inspiration, and defiance in work activities, while absorption is characterized by a state of deep concentration in which individuals feel fully immersed in their work (González-Romá et al., 2006). Empirical research by Yalabik et al. (2017) shows a correlation between job satisfaction and engagement.

Although engagement typically functions as a protective factor, its effects may vary depending on the type of demand encountered. Recent studies grounded in the Challenge–Hindrance–Threat (C–H–T) framework suggest that engagement is promoted by challenge demands that provide growth opportunities. However, they also suggest that it may be undermined by hindrance or threat demands that block goal attainment (Fernandez de Henestrosa et al., 2023; Kim et al. 2025). This highlights the importance of examining engagement as a mediator in contexts of intensifying demands.

Job satisfaction, one of the most extensively studied outcomes in organizational psychology due to its impact on mental health, psychological well-being, and overall life satisfaction among workers (Matud et al., 2024), is defined as a positive emotional state resulting from the evaluation of one’s job and work experiences (Ali, 2016; Robbins and Judge, 2013). This construct encompasses both intrinsic and extrinsic facets of the work environment, including factors such as supervision quality, working conditions, and organizational support.

The empirical evidence on the effect of job demand intensification on engagement is inconsistent. Whereas Burke et al. (2009), Fiksenbaum et al. (2010), Mauno et al. (2024), and Kubicek et al. (2023) report positive associations, especially when intensification takes the form of learning and decision-making demands, others such as Andrade and Neves (2024) and Chouhan (2023) document negative effects when intensification is accompanied by illegitimate tasks or by the erosion of personal time. This inconsistency is consistent with the Challenge–Hindrance–Threat framework (Fernandez de Henestrosa et al., 2023; LePine et al., 2005), which suggests that the same stimulus may operate as a challenge or as a hindrance depending on the worker’s resources. The present study contributes to this discussion by empirically examining, in the Chilean working context, whether job demand intensification exerts a direct negative effect and a positive indirect effect, through engagement, on job satisfaction.

Taken together, the literature suggests a dynamic system in which job insecurity and work–family conflict act as antecedents that intensify job demands, while engagement mediates the link between these intensified demands and job satisfaction. International research has examined these associations, but empirical studies in Latin America remain limited. In Chile, preliminary evidence indicates that work–family conflict has a detrimental effect on satisfaction (Orellana et al., 2023), underscoring the need to examine how emerging psychosocial risks and work engagement jointly influence employees’ well-being in this context.

Our research aligns with Sustainable Development Goal 8 proposed by the United Nations (UN, 2015), which promotes decent work in safe working environments free from discrimination. Chile is currently an emerging economy undergoing accelerated transformations in the organization of work, characteristic of modern working environments (Pavlista et al., 2024), with a regulatory transition toward shorter workweeks (Law 21.561) and weekly working hours that remain above the 40-h recommendation of the International Labour Organization (ILO, 2025). Data from the Superintendence of Social Security show that mental disorders represent a growing share of medical leave, with the work–life balance dimension emerging as one of the factors most strongly associated with elevated mental-health risk (Superintendencia de Seguridad Social, 2024). This setting makes Chile an interesting case in which to study how job demand intensification articulates with job insecurity, work–family conflict and engagement, and allows for testing evidence largely produced in European or Asian contexts.

Building on this evidence, the present study examines how job insecurity, family–work conflict, and work–family conflict contribute to job demand intensification and how engagement mediates the relationship between demand intensification and job satisfaction.

**Figure 1:**
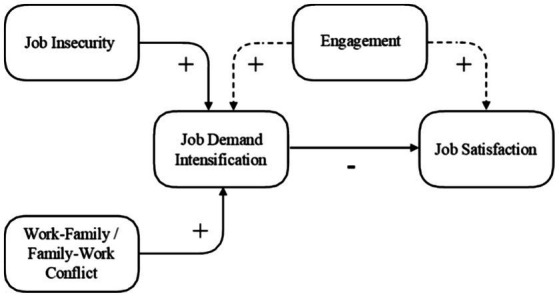
Research model (hypothesized model).

**Figure 2:**
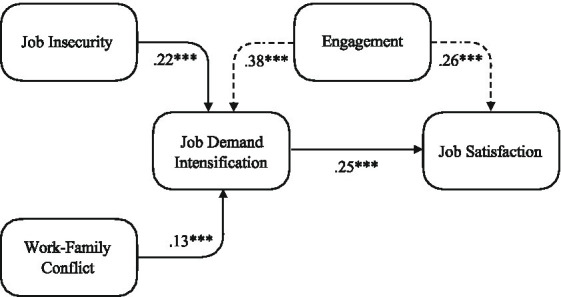
Results of the model for workers in Chile. *p* < 0.001^***^; *p* < 0.01^**^; *p* < 0.05^*^.

The conceptual relationships among the study variables follow an articulated logic: job insecurity and work–family conflict operate as psychosocial antecedents that amplify the subjective perception of job demand intensification. Intensification, in turn, does not produce uniform effects on well-being, since its impact on job satisfaction depends on the motivational resources available (Wang, 2022; Bruck et al., 2002; Gao et al., 2013; Giménez-Espert et al., 2020; Oliveira et al., 2026), among which engagement plays a central role as an intermediate mechanism (Pujol-Cols and Lazzaro-Salazar, 2018). This logic underpins the hypothesized model of the present study and situates previous findings —mainly of European and North American origin— in the Chilean context (see Figures 1, 2).

Specifically, we test the following hypotheses:

*H1*. Perceived job insecurity will be positively associated with job demand intensification (Hunt and Pickard, 2022; Shoss, 2017).

*H2a*. Work-to-family conflict will be positively associated with job demand intensification (Andrade and Neves, 2024; Mansour et al., 2021).

*H2b*. Family-to-work conflict will be positively associated with job demand intensification (Sankar, 2024).

*H3a*. Job demand intensification will have a direct negative effect on job satisfaction (Chouhan, 2023; Mauno et al., 2022).

*H3b*. Engagement will positively mediate the relationship between job demand intensification and job satisfaction, generating a positive indirect effect (Burke et al., 2009; Mauno et al., 2024; Schaufeli and Bakker, 2010).

## 2 Materials and methods

### 2.1 Participants

The final sample comprised 420 workers from Chile. The mean age was 42.4 years (*SD* = 10.73). Women accounted for 62.1% of participants, while men accounted for 37.9%. Marital status was as follows: 35.7% married, 10.2% cohabiting, 40.5% single, 12.9% divorced/separated, and 0.7% widowed. Educational attainment was as follows: 0.95% primary, 9.05% secondary, 10.48% technical, 47.86% university, and 31.67% postgraduate. Sector distribution was 4.3% primary, 26.9% secondary, 29.4% tertiary, 2.4% quaternary, and 36.9% quinary. Sampling was non-probabilistic and convenience-based, consistent with previous organizational studies (Hernández et al., 2000).

The inclusion criteria for participation in the study were as follows: participants had to be 18 years of age or older, currently employed in Chile under a formal employment contract, and willing to provide informed consent prior to participation.

### 2.2 Instruments

All instruments were validated in Spanish versions appropriate for the Chilean context. Internal consistency was estimated with Cronbach’s (*α*), composite reliability ( $\rho_{c}$ ), and average variance extracted (AVE).

*Job insecurity* was assessed using the *job insecurity scale* (JIS-8; Pienaar et al., 2013), adapted to Spanish by Llosa et al. (2017). This includes two subscales: cognitive insecurity (e.g., “I fear that I might get fired”; *α* = 0.90) and affective insecurity (e.g., “I am very sure that I will be able to keep my job”; *α* = 0.78). These were measured using 8 items rated from 1 (“strongly disagree”) to 5 (“strongly agree”).

*Work–family and family–work conflict* were measured with the FWC-WFC/S-10 *scale* (Netemeyer et al., 1996; Blanch, A., and Aluja, A., 2022), validated in Spanish by Schetsche et al. (2022). This has two subscales: the work–family subscale (*α* = 0.88), which comprises 5 items (e.g., “The demands of my work interfere with my home and family life”); and the family–work subscale (*α* = 0.87), which also comprises 5 items (e.g., “The demands of my family interfere with work-related activities”). Responses ranged from 1 (“strongly disagree”) to 7 (“strongly agree”).

*Intensification of job demands* was assessed with the *IDS-15* (Kubicek et al., 2014) adapted to Spanish by Blanco-Donoso et al. (2023), which evaluates five dimensions: (1) Work intensification (e.g., “It is increasingly difficult for me to have enough time for work tasks”); (2) Intensified job-related planning and decision-making demands (e.g., “I increasingly define for myself the way I do work”); (3) Intensified career-related planning and decision-making demands (e.g., “My own professional development increasingly requires keeping other opportunities open”); (4) Intensified knowledge-related learning demands (e.g., “I must acquire new knowledge for my work more often”); and (5) Intensified skills-related learning demands (e.g., “There are increasingly new processes at work that I need to familiarize myself with”). Cronbach’s alphas ranged from 0.71 to 0.85. Items were rated on a scale ranging from 1 (“not at all”) to 5 (“completely”).

*Job Satisfaction* was assessed with the *S10/12* scale (Meliá and Peiró, 1989), which comprises three factors: (1) Satisfaction with supervision (*α* = 0.89; e.g., “The proximity and frequency with which it is supervised”); (2) Satisfaction with the services received (α = 0.72; e.g., “The degree to which your company complies with the agreement, provisions and labor laws”); and (3) Satisfaction with the Physical Environment (α = 0.74; e.g., “The cleanliness, hygiene and health of your workplace”). Items were rated from 1 (“very dissatisfied”) to 7 (“very satisfied”).

Work -Family and Family -Work Conflict were measured with the FWC-WFC/S-10 scale (Netemeyer et al., 1996; Blanch and Aluja, 2009), validated in Spanish by Schetsche et al. (2022). This scale has a one-factor structure with three items: (1) Vigor (e.g., “At my work, I Feel Bursting with Energy”); (2) Dedication (e.g., “I am enthusiastic about my job”; and 3) Absorption (e.g., “I am immersed in my work”). Cronbach’s alpha was 0.81. Responses ranged from 1 (“never”) to 7 (“every day”).

### 2.3 Procedure

For this study, we used a cross-sectional, correlational design. Data were collected through an online self-administered questionnaire distributed via professional networks and organizational contacts. The online modality facilitated access to employees across multiple economic sectors in Chile, ensuring heterogeneity in the sample. Non-probabilistic sampling (Hernández et al., 2000), also known as accidental random or convenience sampling, was used.

The data analyzed in this study were collected in Chile between March and July 2025. A total of 444 questionnaires were initially received. Prior to analysis, cases with incomplete questionnaires or substantial missing data were removed from the database, yielding a final sample of 420 participants.

The study adhered to ethical standards for research with human participants. Ethical approval was granted by the Ethics Committee for Research into People, Society and the Environment (CEIPSA) of Universitat Rovira i Virgili (URV_DPD_24-40_Riesgos_psicosociales_v1.0, OCAS-915). Participation was voluntary, anonymous, and contingent on informed consent.

### 2.4 Data analysis

Data were screened for missing values, outliers, and normality. Missing data were <2% and handled using expectation–maximization procedures. Univariate skewness and kurtosis values were within acceptable thresholds (|2|), thus supporting the use of maximum likelihood estimation with robust corrections. Internal consistency was evaluated by Cronbach’s alpha; all values were ≥0.70.

Measurement Model (CFA) tested the factorial validity of the latent variables. The measurement model was expressed as Equation 1:

$$y=\Lambda_{y}\;\eta +\epsilon ,$$ (1)

where y is the vector of observed indicators, $\Lambda_{y}$ is the factor loading matrix, η represents the latent endogenous variables, and ϵ denotes measurement errors. Convergent validity was assessed through standardized factor loadings (preferably ≥ 0.50, although values ≥0.30 were considered acceptable when theoretically justified and statistically significant), average variance extracted (AVE > 0.50), and composite reliability $(\rho_{c}>0.70).$ Discriminant validity was evaluated using the Fornell–Larcker criterion.

The hypothesized structural equation model was estimated to test the mediating role of engagement. The model was expressed as Equation 2:

η=Bη+Γξ+ζ, (2)

where ξ denotes the latent exogenous variables (job insecurity, work-to-family conflict, and family-to-work conflict), η represents the latent endogenous variables (Job Demand Intensification, Engagement, and Job Satisfaction), B contains the relationships among endogenous variables, Γ contains the effects of exogenous variables on endogenous variables, and ζ represents structural disturbances. Mediation was tested by evaluating the indirect effect (Equation 3):

$$IE_{\mathrm{JDI}\to \mathrm{ENG}\to \mathrm{JS}}=\beta_{\mathrm{ENG},\mathrm{JDI}}\times \beta_{\mathrm{JS},\mathrm{ENG}}$$ (3)

with bootstrap resampling (5,000 samples, 95% confidence intervals). Direct, indirect, and total effects were estimated.

Model fit was evaluated using multiple indices: chi-square (*χ*^2^), degrees of freedom (df), comparative fit index (CFI), Tucker–Lewis index (TLI), root mean square error of approximation (RMSEA) with 90% confidence interval, and standardized root mean square residual (SRMR). Following conventional criteria, CFI ≥ 0.90, TLI ≥ 0.90, RMSEA ≤ 0.08, and SRMR ≤ 0.08 were considered indicative of acceptable fit (Hu and Bentler, 1999).

All analyses were conducted using Python 3.11.11 (Python Software Foundation, 2024) with the *semopy* package (Semopy Developers, 2022) along with *pandas*, *numpy*, *sklearn*, *networkx*, and *matplotlib*. Complementary analyses were performed in R 4.4.3 (R Core Team, 2023) using the widely used *lavaan* package for structural equation modeling (Rosseel, 2012).

## 3 Results

Spearman’s correlation was used to examine the relationships between ordinal variables, and Cronbach’s alpha coefficient was used to evaluate the internal reliability of the measurement instruments. All variables showed positive and statistically significant correlations with each other (*p* < 0.01). The results of the correlation analysis between the variables are presented in Table 1.

**Table 1:** Spearman’s correlation matrix and Cronbach’s alpha coefficient (diagonal row).

Variable	Job insecurity	Work–family conflict	Job demand intensification	Engagement	Job satisfaction
Job insecurity	0.83				
Work–family conflict	0.27***	0.88			
Job demand intensification	0.35***	0.35***	0.82		
Engagement	0.24***	0.13***	0.18***	0.72	
Job satisfaction	0.32***	0.33***	0.35***	0.32***	0.87

Values in the diagonal row represent Cronbach’s alpha. *p* values: *p* < 0.001^***^; *p* < 0.01^**^; *p* < 0.05^*^.

The observed correlations satisfy the assumptions required to perform the hypothesis test. Table 2 presents the fit indices of the proposed mediation model, which evaluates the mediating role of engagement in the relationship between job demand intensification and job satisfaction.

**Table 2:** Mediating role of engagement between work demand intensification and job satisfaction among workers in Chile (*n* = 420).

*χ* ^2^	df	CFI	GFI	AGFI	NFI	TLI	RMSEA	AIC	BIC
2463.31	854	0.76	0.68	0.66	0.68	0.75	0.06	172.26	543.97

CFI, comparative fit index; GFI, goodness-of-fit index; AGFI, adjusted goodness-of-fit index; NFI, normed fit index; TLI, Tucker–Lewis index; RMSEA, root mean square error of approximation; AIC, Akaike information criterion; BIC, Bayesian information criterion.

Model fit was evaluated using multiple indices: chi-square (χ²), degrees of freedom (df), Comparative Fit Index (CFI), Tucker -Lewis Index (TLI), Root Mean Square Error of Approximation (RMSEA) with 90% confidence interval, and Standardized Root Mean Square Residual (SRMR). The evaluation of model fit followed classical and contemporary recommendations for covariance structure analysis (Bentler and Bonett, 1980; Hu and Bentler, 1999), the incremental fit indices (CFI = 0.76; TLI = 0.75) remained substantially below the cut-off values suggested in the literature (Bentler, 1990). This indicates that the model did not fully meet conventional global fit criteria. Accordingly, these results should be interpreted with caution when assessing the explanatory capacity of the mediation model in the studied sample of Chilean workers. This methodological limitation, along with its implications, is further discussed in the Limitations section.

Table 3 presents the direct and indirect effects of the model variables on the relationships among work demand intensification, engagement, work–family conflict, job insecurity, and job satisfaction. Regarding work–family and family–work conflicts, our results show that only the work–family conflict is statistically significant and is associated with increased work demand intensification. Our results also suggest that work demand intensification, engagement, and the work–family conflict are strongly correlated.

**Table 3:** Direct and indirect effects of the model variables.

Endogenous	Predictor	Effect type	Estimate (Unstd)	Std. Err	Estimate (Std)	*p*-value	Significance
2	Engagement	Job demand intensification	~	0.38	0.11	0.25	0.00057^*^
5	En_1	Engagement	~	1.00	–	0.56	nan
6	En_2	Engagement	~	1.41	0.18	0.79	0.00000^*^
7	En_3	Engagement	~	0.86	0.11	0.49	0.00000^*^
20	Fw_10	Work–family conflict	~	0.91	0.06	0.71	0.00000^*^
16	Fw_6	Work–family conflict	~	1.00	–	0.77	nan
17	Fw_7	Work–family conflict	~	1.04	0.06	0.81	0.00000^*^
18	Fw_8	Work–family conflict	~	1.04	0.06	0.81	0.00000^*^
19	Fw_9	Work–family conflict	~	1.00	0.06	0.78	0.00000^*^
21	Ids_1	Job demand intensification	~	1.0	–	0.37	nan
30	Ids_10	Job demand intensification	~	1.66	0.24	0.62	0.00000^*^
31	Ids_11	Job demand intensification	~	1.58	0.23	0.59	0.00000^*^
32	Ids_12	Job demand intensification	~	1.68	0.24	0.63	0.00000^*^
33	Ids_13	Job demand intensification	~	1.81	0.25	0.68	0.00000^*^
34	Ids_14	Job demand intensification	~	1.71	0.24	0.64	0.00000^*^
35	Ids_15	Job demand intensification	~	1.44	0.22	0.54	0.00000^*^
22	Ids_2	Job demand intensification	~	0.92	0.17	0.34	0.00000^*^
23	Ids_3	Job demand intensification	~	1.06	0.18	0.40	0.00000^*^
24	Ids_4	Job demand intensification	~	1.03	0.18	0.38	0.00000^*^
25	Ids_5	Job demand intensification	~	0.97	0.18	0.36	0.00000^*^
26	Ids_6	Job demand intensification	~	1.12	0.19	0.42	0.00000^*^
27	Ids_7	Job demand intensification	~	1.02	0.18	0.38	0.00000^*^
28	Ids_8	Job demand intensification	~	1.15	0.19	0.43	0.00000^*^
29	Ids_9	Job demand intensification	~	1.02	0.18	0.38	0.00000^*^
0	Job demand intensification	Job insecurity	~	0.21	0.05	0.30	0.00002^*^
1	Job demand intensification	Work–family conflict	~	0.12	0.03	0.26	0.00006^*^
8	JIS_1	Job Insecurity	~	1.00	–	0.52	nan
9	JIS_2	Job insecurity	~	0.61	0.10	0.32	0.00000^*^
10	JIS_3	Job insecurity	~	0.84	0.11	0.44	0.00000^*^
11	JIS_4	Job insecurity	~	1.10	0.12	0.58	0.00000^*^
12	JIS_5	Job insecurity	~	1.50	0.14	0.79	0.00000^*^
13	JIS_6	Job insecurity	~	1.18	0.12	0.62	0.00000^*^
14	JIS_7	Job insecurity	~	1.63	0.14	0.86	0.00000^*^
15	JIS_8	Job insecurity	~	1.30	0.13	0.68	0.00000^*^
36	SL_1	Job satisfaction	~	1.00	–	0.37	nan
45	SL_10	Job satisfaction	~	2.04	0.27	0.75	0.00000^*^
46	SL_11	Job satisfaction	~	1.67	0.24	0.62	0.00000^*^
47	SL_12	Job satisfaction	~	1.51	0.22	0.56	0.00000^*^
37	SL_2	Job satisfaction	~	1.15	0.19	0.42	0.00000^*^
38	SL_3	Job satisfaction	~	1.40	0.21	0.52	0.00000^*^
39	SL_4	Job satisfaction	~	0.94	0.17	0.34	0.00000^*^
40	SL_5	Job satisfaction	~	2.12	0.28	0.78	0.00000^*^
41	SL_6	Job satisfaction	~	2.11	0.28	0.78	0.00000^*^
42	SL_7	Job satisfaction	~	1.96	0.27	0.72	0.00000^*^
43	SL_8	Job satisfaction	~	1.92	0.26	0.71	0.00000^*^
44	SL_9	Job satisfaction	~	1.77	0.25	0.65	0.00000^*^
4	Job satisfaction	Job demand intensification	~	0.25	0.06	0.25	0.00028^*^
3	Job satisfaction	Engagement	~	0.25	0.05	0.39	0.00000^*^

*Indicates statistical significance at *p* < 0.001.

Job satisfaction and job insecurity are also associated with these factors, which supports the hypothesis of a mediation model. Demand intensification directly affects job satisfaction, indicating that work pressures can also directly influence perceptions of satisfaction.

Regarding the indirect effects, engagement positively influenced job satisfaction, thereby mediating the relationship between work demand intensification and job satisfaction. This mediation suggests that, despite intensified work demands, engagement functions as a protective resource that fosters job satisfaction.

Table 4 reports the measurement model parameters. Overall, the indicators showed statistically significant standardised factor loadings of moderate to high magnitude, providing support for the latent constructs included in the model. Factor loadings ranged from 0.32 to 0.86. The highest values were observed for the work–family conflict and job satisfaction constructs, while some indicators of job demand intensification and job insecurity presented moderate loadings.

**Table 4:** Measurement model parameters.

Construct	Indicator	Standardised loading (β)	p− value
Engagement	En_1	0.56	__
	En_2	0.79	<0.001
	En_3	0.50	<0.001
Work–family conflict	Fw_6	0.77	__
	Fw_7	0.81	<0.001
	Fw_8	0.81	<0.001
	Fw_9	0.78	<0.001
	Fw_10	0.71	<0.001
Job demand intensification	Ids_1	0.37	__
	Ids_2	0.34	<0.001
	Ids_3	0.40	<0.001
	Ids_4	0.38	<0.001
	Ids_5	0.36	<0.001
	Ids_6	0.42	<0.001
	Ids_7	0.38	<0.001
	Ids_8	0.43	<0.001
	Ids_9	0.38	<0.001
	Ids_10	0.62	<0.001
	Ids_11	0.59	<0.001
	Ids_12	0.63	<0.001
	Ids_13	0.68	<0.001
	Ids_14	0.64	<0.001
	Ids_15	0.54	<0.001
Job insecurity	JIS_1	0.52	__
	JIS_2	0.32	<0.001
	JIS_3	0.44	<0.001
	JIS_4	0.58	<0.001
	JIS_5	0.79	<0.001
	JIS_6	0.62	<0.001
	JIS_7	0.86	<0.001
	JIS_8	0.68	<0.001
Job satisfaction	SL_1	0.37	__
	SL_2	0.42	<0.001
	SL_3	0.52	<0.001
	SL_4	0.34	<0.001
	SL_5	0.78	<0.001
	SL_6	0.78	<0.001
	SL_7	0.72	<0.001
	SL_8	0.71	<0.001
	SL_9	0.65	<0.001
	SL_10	0.75	<0.001
	SL_11	0.62	<0.001
	SL_12	0.56	<0.001

Table 5 reports the estimated structural relationships among the latent variables of the SEM. Job insecurity ( β = 0.30, p < 0.001) and work–family conflict ( β = 0.26, p < 0.001) were positively and significantly associated with job demand intensification. Job demand intensification, in turn, showed positive and significant effects on both engagement ( β = 0.25, p < 0.001) and job satisfaction ( β = 0.25, p < 0.001). Finally, engagement displayed a positive and significant effect on job satisfaction ( β = 0.39, p < 0.001), representing the largest structural effect within the model.

**Table 5:** Structural relationships of the SEM.

Endogenous variable	Predictor	Standardised β	Estimate (Std)	*p*-value
Engagement	Job demand Intensification	0.25	0.11	<0.001
Job demand intensification	Job insecurity	0.30	0.05	<0.001
Job demand intensification	Work–family conflict	0.26	0.03	<0.001
Job satisfaction	Job demand intensification	0.25	0.06	<0.001
Job satisfaction	Engagement	0.39	0.05	<0.001

*β*: standardized regression coefficient. SE: standard error. *p* values: *p* < 0.001^***^; *p* < 0.01^**^; *p* < 0.05^*^.

As reported in Table 6, job demand intensification showed both a direct effect on job satisfaction ( β = 0.25, p < 0.001) and an indirect effect mediated by engagement ( β = 0.10, p < 0.001). The positive indirect effect suggests that higher levels of job demand intensification can increase engagement, which in turn fosters higher levels of job satisfaction. Taken together, the findings support a partial mediation effect of engagement in the relationship between job demand intensification and job satisfaction.”

**Table 6:** Direct and indirect effects of the model.

Relationship	Type of effect	Standardised β	p− value
Job demand intensification → job satisfaction	Direct	0.25	<0.001
Job demand intensification → engagement	Direct	0.25	<0.001
Engagement → job satisfaction	Direct	0.39	<0.001
Job demand intensification → engagement → job satisfaction	Indirect	0.10	<0.001

*β*: standardized regression coefficient. *p* values: *p* < 0.001^***^; *p* < 0.01^**^; *p* < 0.05^*^.

This study has examined the mediating role of work engagement in the relationship between job demand intensification and job satisfaction, with job insecurity and work–family conflict as antecedents. Results have shown that both job insecurity and work–family conflict significantly predicted the perceived intensification of job demands. In turn, job demand intensification showed a positive direct association with job satisfaction and, additionally, a positive indirect effect through engagement. Both pathways converge in the same direction but capture different psychological dimensions, as we discuss below.

Our first hypothesis is fully confirmed. Our results indicate that job insecurity directly intensifies work demands. Hunt and Pickard (2022), for their part, indicate that job insecurity associated with changes in valued job characteristics, such as reduced pay or unexpected changes in work schedules, increases the likelihood of working with a high degree of intensity. On the other hand, Hünefeld et al. (2025) reported that women and highly qualified employees experience higher levels of work intensity and work intensification, and that restructuring involving new learning requirements, increased responsibilities, or the incorporation of new management practices leads to higher work intensity. According to Mauno and Kinnunen (2021), work demand intensification has detrimental consequences for employees’ well-being and motivation. It has also been suggested that this intensification correlates with other work stressors, such as job insecurity and work–family conflict.

The second hypothesis is partially supported. H2a was supported, work-to-family conflict was positively associated with job demand intensification. H2b was not supported, family-to-work conflict did not show a statistically significant effect on intensification in this sample. This asymmetric pattern is consistent with COR theory, which predicts that the directional spillover from work to family—more than the reverse—consumes the time and energy resources that workers would otherwise mobilize to cope with their job tasks. Andrade and Neves (2024) suggested that the intensification of work had a direct, positive effect on work–family conflict, and that work–family conflict also had a direct, negative effect on engagement. In this study, work–family conflict was positively associated with the intensification of work demands. Mansour et al. (2021) found that one factor behind the intensification of work is the use of smartphones, tablets, and other devices outside working hours, which blurs boundaries and generates more work–family conflict. It has also been reported that workers with a high level of autonomy are potentially exposed to work demand intensification (Kubicek et al., 2017) owing both to the pace of work and to multitasking, which is supported by the use of ICTs (Santarpia et al., 2021). Chouhan (2023) indicates that a high level of work demand intensification negatively affects employees’ well-being, as they are more likely to spend less time with their friends and family. Along these lines, Kubicek et al. (2014) reported that relentlessly demanding jobs that provide no respite from an intense workload affect workers’ health by increasing the potential for stress and exhaustion while negatively affecting their relationships with their family.

Our third hypothesis was partially supported: H3b was confirmed, but H3a was not supported because intensification showed a positive, rather than negative, direct effect on satisfaction. Regarding the indirect path (H3b), engagement positively mediated the relationship between intensification and satisfaction, confirming its role as a motivational resource. This finding aligns with prior evidence, Burke et al. (2009) showed that employees with higher work intensity also reported higher engagement and satisfaction, and Kubicek et al. (2013) found that intensification linked to new knowledge and technological acceleration was associated with higher engagement and lower burnout. Consistently, Mauno et al. (2024) reported that intensification of planning and decision-making demands was associated with higher engagement, and Fiksenbaum et al. (2010) documented a positive association between work intensity and engagement. The opposite has also been documented when intensification involves illegitimate tasks that undermine meaning at work and threaten professional identity (Andrade and Neves, 2024).

Regarding the direct path, the positive association between intensification and satisfaction, together with the positive indirect effect through engagement, suggests that demands in this context operated predominantly as challenge demands: taxing, yet perceived as opportunities for growth and accomplishment (LePine et al., 2005; Bakker and Demerouti, 2017; Mauno et al., 2024). This reading is consistent with evidence that learning, planning and decision making demands act as positive predictors of work well being when workers have sufficient autonomy and self-efficacy (Kubicek et al., 2023).

This pattern nonetheless contrasts with much of the evidence linking intensification with exhaustion, stress and lower satisfaction (Andrade and Neves, 2024; Chouhan, 2023). Three factors may help account for the divergence. First, the high educational level of our sample, historically associated with greater tolerance toward cognitive demands experienced as challenges. Second, the sectoral composition, with a predominance of tertiary and quinary sectors where demands tend to be cognitive rather than physical. Third, the Chilean context of recent regulatory transition, in which perceived intensification may coexist with optimistic expectations about improvements in working conditions. Rather than invalidating the theoretical reasoning behind a negative direct effect, this pattern delimits the conditions under which intensification can be appraised as a challenge. These results should therefore be interpreted with caution and replicated in samples with different sociolabor compositions before being generalized. These findings align with previous research suggesting that psychosocial risks, such as insecurity and work–family conflict, amplify perceptions of workload and time pressure (De Witte et al., 2016; Mansour et al., 2021). Consistent with evidence that engagement mediates the relationship between job demands and well-being outcomes, they also confirm that engagement can act as a protective factor (Schaufeli and Bakker, 2010).

The present study articulates in a complementary manner the Conservation of Resources Theory (COR; Hobfoll, 1989) and Within the Job Demands -Resources (JD-R) model, and depending on available resources, demand intensification can function either as a hindrance, leading to strain, or as a challenge, stimulating growth and engagement (Bakker and Demerouti, 2007; Crawford et al., 2010; Güngör and Sönmez, 2025). From the COR perspective, job insecurity and work–family conflict represent threats to valued resources—such as job stability, economic stability and energy—, which activate processes of resource loss and anticipatory resource consumption to cope with the situation (Hobfoll, 1989; Shoss, 2017). This logic explains why both antecedents amplify the perception of intensified demands. From the JD-R model, intensified job demands are not inherently detrimental: their impact depends on the motivational resources available to each person. Thus, engagement acts as a mediating motivational mechanism that channels demanding job conditions—through vigor, dedication and absorption (Hakanen and Schaufeli, 2012; Schaufeli and Bakker, 2010; Schaufeli et al., 2002)—toward favorable outcomes such as job satisfaction. The complementarity of both frameworks lies in that COR provides the loss/threat component explaining the origin of perceived intensification, while JD-R provides the motivational component explaining how that intensification can be transformed, mediated by engagement, into job satisfaction (González-Romá et al., 2006).

## 4 Discussion

This study provides partial support for the proposed mediation model, although the global fit indices were suboptimal (CFI = 0.76; TLI = 0.75). Specifically, job insecurity and work–family conflict emerged as significant antecedents of job demand intensification, which in turn was positively associated with job satisfaction both directly and indirectly through engagement. Engagement appeared to function as an important motivational mechanism linking intensified demands to more favorable attitudinal outcomes. Therefore, the substantive findings should be interpreted cautiously, as the theoretical relationships were supported despite limitations in overall model fit. These results are consistent with the view that job demands are not inherently detrimental, but that their impact depends on the resources available to employees (Bakker and Demerouti, 2007; Schaufeli and Bakker, 2010).

By testing this model in a Chilean sample, this study helps to address a gap in the literature on emerging psychosocial risks at work within the Chilean population and Latin American organizational psychology. Previous evidence has highlighted the need to contextualize psychosocial risks within specific socio-cultural environments (Orellana et al., 2023). Our findings confirm the cross-cultural applicability of the JD-R and COR frameworks, while emphasizing engagement as a key psychological mechanism linking risks and satisfaction.

Despite the growing body of evidence on job intensification and engagement, some gaps remain. First, most studies examine these relationships in a fragmented manner (Öhman et al., 2026; Mauno et al., 2024; Scheel et al., 2023). Second, the JD-R and COR frameworks are not usually articulated in a complementary way: COR explains why job insecurity and work–family conflict operate as threats that amplify the perception of demands, whereas JD-R explains why engagement can transform those intensified demands into satisfaction. Third, the available evidence comes mainly from Europe and Asia; the Chilean context—characterized by working hours above ILO recommendations, a high prevalence of medical leave for mental disorders associated with work stress (Superintendencia de Seguridad Social, 2024), and a recent regulatory transition (Law 21.561, reducing the workweek)— offers a setting in which this model can be tested. The contribution of the study lies, therefore, in integrating these elements in a single model and empirically testing it in a Latin American emerging economy.

It is important to distinguish the direct effect of job intensification on job satisfaction, which reflects a positive cognitive appraisal of demands as stimulating challenges, from the indirect effect through engagement, which reflects a sustained motivational process, vigor, dedication and absorption, that channels this initial appraisal into durable satisfaction. Although both effects are positive, they capture different dimensions: one cognitive evaluative and the other motivational affective.

Taken together, the results underscore the importance of developing organizational practices that simultaneously reduce job insecurity and work–family conflict, while strengthening engagement. Such practices may enhance employees’ satisfaction and foster healthier, more sustainable workplaces (García-Salirrosas et al., 2023).

Another practical implication is that organizations should provide adequate physical and psychosocial conditions, since these foster higher levels of satisfaction, motivation, and commitment among employees (Dumitriu et al., 2025).

Identifying work characteristics and conditions that promote engagement can enhance employee satisfaction and positively influence organizational performance and achievement of objectives (Yalabik et al., 2013).

Understanding the mediating role of engagement between job demand intensification and job satisfaction challenges organizations to develop practices that strengthen this positive work state. In this context, it becomes essential to promote empowering leadership styles that create and offer job resources that enhance employees’ psychological capital, enabling them to cope with increasing demands and job insecurity (Kim et al., 2025). Leaders play a strategic role in identifying aspects of work most sensitive to perceptions of insecurity, thereby facilitating the development of communication strategies to reduce ambiguity, prevent rumors, and mitigate feelings of vulnerability associated with highly valued job characteristics. In line with this, organizations can implement measures to ensure open communication channels, fostering continuous dialogue and minimizing employee uncertainty.

Balancing work and family is a significant challenge. Individuals should establish boundaries to disconnect from work and digital technologies, thereby creating opportunities for family interaction and self-care. At the organizational level, this necessitates developing policies and norms governing connection and disconnection, as well as promoting activities involving families and fostering a culture of work–family balance. Additionally, organizations should implement training initiatives to enhance employees’ time- and stress-management skills.

The implications derived from this study should be read considering the sample characteristics —mostly women with a high educational level, distributed across tertiary and quinary sectors— and the current Chilean context. Given that work–family conflict has been identified by Superintendencia de Seguridad Social (2024) as a critical psychosocial risk factor in Chile, organizational and people-management policies should prioritize the right to digital disconnection and parental flexibility agreements, both particularly relevant for women workers. Together with the implementation of policies that foster work–life balance, these measures have the potential to improve employee well-being and, above all, to contribute to broader social goals such as gender equality, family stability, and the development of a more sustainable society. However, as Geraldes et al. (2024) show, the mere availability of work–family reconciliation practices is not, in itself, an indicator of organizational success: it depends on the support of supervisors and managers and on perceived organizational support. Leaders who exhibit supportive behaviors toward work–family balance promote greater use of these practices and, consequently, reduce conflict (Oliveira et al., 2026; Fang et al., 2025).

From an ergonomic perspective, these results can inform the design of working conditions and job demands that align with workers’ resources. Understanding how these issues interact will promote job satisfaction and well-being. Along these lines, Dumitriu et al. (2025) suggested that organizations that offer adequate physical (i.e., workplace environment-related) and psychosocial conditions generate greater levels of satisfaction, motivation, and commitment among their employees.

In line with the Economic Commission for Latin America and the Caribbean (CEPAL, 2024), our findings reinforce the need to understand psychosocial risks from a systemic and multilevel perspective, linked to the macroeconomic conditions of the region, characterized by low economic growth. In this sense, job insecurity does not respond solely to an individual perception, but also to an economic environment that pressures workers to accept increasing demands to keep their jobs.

The cross-sectional design of this study precludes causal inferences, and reliance on self-report data may introduce common-method bias. The use of a non-probabilistic sample also limits the generalizability of findings. Future research should use longitudinal or experimental designs to capture the dynamic interplay between job demands, engagement, and satisfaction over time (Bakker and Demerouti, 2017; LePine et al., 2005). It would also be valuable to include multi-source data (e.g., supervisor ratings and objective performance indicators) and examine organizational outcomes such as absenteeism, turnover, and productivity.

Since this study employed a cross-sectional design, causal relationships among the variables analyzed cannot be established. Future longitudinal or experimental research is needed to examine how job insecurity, work–family conflict, and engagement dynamically influence job satisfaction over time (Bakker and Demerouti, 2017; LePine et al., 2005). Secondly, the study’s reliance on self-reported data may have introduced common method variance, though validated instruments were used to mitigate this risk. Thirdly, the use of non-probabilistic sampling limits the generalizability of these findings beyond the study participants. Expanding the sample to include diverse organizational contexts and industries would strengthen external validity. In addition, the measurement and structural model did not fully satisfy conventional global fit criteria, particularly with respect to the incremental fit indices (CFI = 0.76; TLI = 0.75). Although the RMSEA (0.06) indicated acceptable approximate fit, these results suggest that the model only received partial empirical support and that the proposed latent structure may require refinement in future studies.

Despite these limitations, this study provides valuable insights for theory and practice. Our findings highlight the importance of engagement as a psychological mechanism that transforms intensified demands into positive outcomes, thereby extending the explanatory power of the JD-R and COR frameworks in a Latin American context. For organizations, they suggest that reducing perceptions of job insecurity and work–family conflict, while actively promoting engagement, can enhance job satisfaction and foster healthier and more sustainable work environments. Practical strategies include transparent communication, supportive leadership, and policies that promote work–life balance (García-Salirrosas et al., 2023).

While these findings are valuable, future research could examine other forms of working arrangements, such as independent or non-permanent workers, teleworkers or remote employees, and those with extended working hours, as suggested by Adams-Prassl et al. (2020).

This study also has important practical implications. Organizations should seek to minimize employees’ perceptions of job insecurity through transparent communication and stable employment practices, since previous research indicates that insecurity is strongly linked to lower satisfaction and commitment (Jiang and Lavaysse, 2018; De Witte et al., 2016). Moreover, consistent with recent findings in remote work contexts (García-Salirrosas et al., 2023; Lyzwinski, 2024), interventions promoting work–life balance and family-supportive supervisory behaviors can help mitigate work–family conflict. At the same time, initiatives aimed at fostering engagement—such as granting autonomy, providing feedback, and supporting professional development—may enable employees to reinterpret intensified demands as opportunities for growth rather than threats (Bakker and Demerouti, 2017; LePine et al., 2005).

Overall, this study offers partial empirical support for the hypothesized relationships among job insecurity, work–family conflict, job demand intensification, engagement, and job satisfaction. While the results are theoretically consistent with the JD-R and COR frameworks, the suboptimal global fit indices indicate that the model should be regarded as a promising but preliminary representation of these relationships in the Chilean context.

## Data Availability

The raw data supporting the conclusions of this article will be made available by the authors, without undue reservation.
